# Investigating Attitudes, Motivations and Key Influencers for COVID-19 Vaccination Uptake among Late Adopters in Urban Zimbabwe

**DOI:** 10.3390/vaccines11020411

**Published:** 2023-02-10

**Authors:** Azure Tariro Makadzange, Patricia Gundidza, Charles Lau, Janan Dietrich, Nellie Myburgh, Nyasha Elose, Wilmot James, Lawrence Stanberry, Chiratidzo Ndhlovu

**Affiliations:** 1 Charles River Medical Group, 155 King George Avenue, Avondale, Harare, Zimbabwe; 2 GeoPoll, Washington, DC 20036, USA; 3 Perinatal HIV Research Unit (PHRU), Faculty of Health Sciences, University of the Witwatersrand, Johannesburg 2000, South Africa; 4 African Social Sciences Unit of Research and Evaluation (ASSURE), Division of the Wits Health Consortium, Faculty of Health Sciences, University of the Witwatersrand, Johannesburg 2000, South Africa; 5 Health Systems Research Unit, South African Medical Research Council, Bellville 7530, South Africa; 6 Wits Vaccines & Infectious Diseases Analytics (VIDA) Research Unit, Faculty of Health Sciences, University of the Witwatersrand, Johannesburg 2000, South Africa; 7 Institute for Social and Economic Research and Policy, Columbia University, IAB 118th Street, New York, NY 10025, USA; 8 Vaccine Information Network, Columbia University, 533 W 218th St., New York, NY 10032, USA; 9 Department of Pediatrics, Vagelos College of Physicians and Surgeons, Columbia University, New York, NY 10032, USA; 10 Internal Medicine Unit, Faculty of Health Sciences, University of Zimbabwe, Harare, Zimbabwe

**Keywords:** COVID-19 vaccination, vaccine hesitancy, vaccine uptake in Africa, COVID-19 vaccines

## Abstract

The rapid development of vaccines in response to the COVID-19 pandemic has provided an effective tool for the management of COVID-19. However, in many African countries there has been a poor uptake of COVID-19 vaccines with only 32.5% first vaccine dose coverage compared to the WHO global target of 70%. As vaccine access improves, one of the important drivers of low uptake has been vaccine hesitancy, driven by levels of confidence, convenience, and complacency. Between 4 January–11 February 2022, we conducted a survey of vaccine late adopters to assess factors that influenced adults in Harare, Zimbabwe to present for their first COVID-19 vaccine dose almost 12 months after the vaccination program began. Of the 1016 adults enrolled, 50% were female and 12.4% had HIV co-infection. Binary logistic regression models were developed to understand factors associated with vaccine confidence. Women were more likely to have negative views about the COVID-19 vaccine compared to men (OR 1.51 (95%CI 1.16, 1.97, *p* = 0.002). Older adults (≥40 years) compared with youth (18–25 years) were more likely to have ‘major concerns’ about vaccines. When asked about their concerns, 602 (59.3%) considered immediate side effects as a major concern and 520 (52.1%) were concerned about long-term health effects. People living with HIV (PLWH) were more likely to perceive vaccines as safe (OR 1.71 (95%CI: 1.07, 2.74, *p* = 0.025) and effective (1.68 (95%CI: 1.07, 2.64, *p* = 0.026). Internet users were less likely to perceive vaccines as safe (OR 0.72 (95% CI: 0.55, 0.95, *p* = 0.021) compared to non-Internet users; and social media was a more likely source of information for youth and those with higher education. Family members were the primary key influencers for 560 (55.2%) participants. The most important reason for receiving the COVID-19 vaccine for 715 (70.4%) participants was the protection of individual health. Improving vaccine coverage will need targeted communication strategies that address negative perceptions of vaccines and associated safety and effectiveness concerns. Leveraging normative behavior as a social motivator for vaccination will be important, as close social networks are key influences of vaccination.

## 1. Introduction

The severe acute respiratory syndrome coronavirus 2 (SARS-CoV-2) has had a devastating impact on health and socio-economic well-being for millions of people. Since the start of the pandemic, there have been over 620 million cases and over 6.5 million deaths [1]. Vaccines are one of the most effective approaches for the public health management of many infectious diseases. In response to the SARS-CoV-2 pandemic, over 300 vaccines have gone into development across various platforms [2]. Among these, 35 have been approved by at least one country and 11 have been approved by the World Health Organization (WHO) [3]. In addition to manufacturing and distribution, widespread vaccination acceptance will be required to achieve sufficient vaccination coverage rates [4].

In pre-pandemic 2019, the WHO identified vaccine hesitancy as one of the top 10 threats to human health [5]. The WHO Strategic Advisory Group of Experts (SAGE) on immunization has defined vaccine hesitancy as a behavior influenced by convenience, confidence, and complacency towards vaccines [6]. Factors that influence and drive vaccine hesitancy are often complex and context-specific [7]. The SAGE working group has established a model of determinants of hesitancy that focuses on three main domains that evaluate contextual influences, individual and group influences, and vaccine and vaccination-specific influences [6]. The contextual influences include socio-demographic, cultural, economic, health systems and political factors. In infant vaccinations, for example, parental approval plays an important role and may be heavily influenced by socio-economic factors, including the father’s education [8]. In infant BCG vaccination uptake in Nigeria, maternal factors such as immunization knowledge, health-seeking practices and social influence were important predictors of uptake [9]. In pediatric influenza vaccination in Kenya, distance from vaccination center, maternal age, impact of time off for vaccination and household experience with severe influenza were important factors in driving vaccine uptake [10]. In Africa, adult vaccinations are not part of routine primary care, and much less is known about determinants of hesitancy for adult vaccines. COVID-19 vaccination is among the first efforts within Africa for adult vaccination. 

Vaccine hesitancy in Africa is poorly understood, particularly for adult vaccines and within key subpopulations, such as people living with HIV (PLWH) [11]. Studies conducted prior to the availability of COVID-19 vaccines suggested that vaccine acceptance rates would be high in Africa [12]. However, as vaccine access improves, it has become increasingly evident that vaccine hesitancy was a major driver of low COVID-19 vaccine coverage rates in Africa [13]. Vaccine hesitancy lies on a spectrum that includes early adopters, those who take a ‘wait and see’ approach, and those who do not accept all vaccines. Current efforts to improve vaccine uptake should focus on those that have taken a ‘wait and see’ approach, as they have the potential to become late adopters. There is limited characterization concerning late adopters in Africa. 

The WHO set a global goal of achieving 70% COVID-19 vaccination coverage by mid-2022 [14]. However, by early 2022, Africa had less than 15% vaccine coverage with vaccine hesitancy being an important driver of low coverage rates [15]. One year after initiating its vaccination program, vaccine uptake slowed and first dose coverage in Zimbabwe was 45.5% and second dose coverage was 35.4% (MOHCC, Zimbabwe), well below global coverage goals [13]. We conducted a survey among individuals who were presenting for their first COVID-19 vaccine at public vaccination centers in Harare, Zimbabwe almost one year after the initiation of the national vaccination program. We defined these individuals as ‘wait and seers’ who had become late adopters [6]. Defining attitudes, barriers and motivations can inform the design of interventions to promote vaccine uptake. 

The aim of this study was to measure the environmental and individual factors (attitudes, barriers, motivations, key influencers, and information sources) that influence vaccine uptake among late adopters [16]. The key gap addressed by this study was the lack of real-time data on factors affecting vaccine uptake in urban African populations, such as those in Harare, Zimbabwe, at a time when access was no longer a major barrier. 

## 2. Materials and Methods

### 2.1. Study Design

Between 4 January and 11 February 2022, we conducted a quantitative survey through face-to-face interviews. 

### 2.2. Study Sites and Sampling

Eligible adults (≥18 years) who were receiving their first dose of COVID-19 vaccines at public sites providing free vaccine services at four City of Harare clinics (Wilkins, Kuwadzana, Budiriro, Mabvuku), including their affiliated outreach sites (Mabelreign, Avondale, and Dzivarasekwa), were consecutively enrolled into the study. The clinics were large polyclinics distributed across the city as well as their affiliated mobile outreach units. All eligible adults receiving their first COVID-19 vaccine were eligible to participate except those who had obvious cognitive impairments or were unable to provide informed consent. A sample size of 1000 participants was considered to be sufficiently large to reach saturation in key themes related to attitudes, motivations and barriers to vaccination. 

### 2.3. Study Procedures

Participants at each site who were receiving the first dose of the vaccine were informed about the study while queueing to receive the vaccine. Those that agreed and were eligible were recruited, an informed consent form signed, and they participated in the survey after receiving their vaccination. Participants were reimbursed with $5 for participating in the survey.

### 2.4. Study Measures

A comprehensive questionnaire was prepared based on a user-centered design framework [17]. The questionnaire consisted of six main sections including demographics (sex, age, education, socio-economic status), attitudes and views towards COVID-19 vaccines, barriers, motivations, information sources and vaccination experience. Age was categorized into three groups: ages 18–25 as youth, 26–39 years as young adults and ≥40 years as older adults. Vaccine confidence was measured through survey statements related to individual perception on safety, effectiveness of vaccines and confidence in country’s regulatory approval process for vaccines [18]. Socioeconomic status was based on an assessment of available resources—electricity, television, refrigerator, bed, battery, or generator for power. High socioeconomic status was defined by owning both a battery or a generator for power and a refrigerator, low economic status was defined by owning neither a refrigerator or an alternative source of power. In addition, Internet usage in the last 30 days, was assessed.

### 2.5. Statistical Analysis

Data were analyzed using Stata version 13 (College Station, TX 77845, USA). Baseline socio-demographic data and responses to survey questions were summarized using means and standard deviations (SD) for normally distributed data or medians and interquartile ranges (IQRs) for non-normally distributed continuous variables. Absolute numbers, proportions and percentages were used for categorical data. The chi-squared test was used to test for significance between outcome variables and demographic variables such as gender, age, education, economic status, previous personal experience with COVID-19, HIV status, and Internet use over the last 30 days. 

Binary logistic regression models were developed to examine factors associated with ‘very negative’ and ‘somewhat negative’ views towards vaccines when they were first available, as well as concerns about vaccines. The outcome variables were dichotomized (Very negative/somewhat negative or Neutral/somewhat positive/very positive; Major concerns or Minor concerns/No concerns; Strongly agree or somewhat agree/neutral/somewhat disagree/strongly disagree). The variables evaluated included demographic factors such as gender, age, education, and income (a proxy for which was defined by availability of alternate sources of energy (battery or generator), and/or refrigerator ownership), personal experience with COVID-19—knowing someone who became severely ill or died from COVID-19, Internet usage and HIV status. *p*-values were two-sided.

### 2.6. Ethics Approvals

The protocol, consent forms and recruitment materials were reviewed and approved by the Medical Research Council of Zimbabwe, Joint Research Ethics Committee (JREC) of Parirenyatwa Hospital, the University of Zimbabwe, and Harare City Health Departments prior to initiation of the study. All amendments to the protocol, consent forms and/or recruitment materials were approved by the respective institutional review boards before they were implemented. All participants provided written informed consent. 

## 3. Results

A total of 1016 adults were enrolled into the study; 1013 (99.7%) received the Sinopharm vaccine and three (0.3%) received the Sinovac vaccine. Gender was equally distributed in the cohort with 508 (50%) females, and a median age of 30 years (IQR 22–39) (Table 1). All participants were of African descent, and most had attained high school education. The self-reported comorbid conditions were diabetes (1.5%), cardiac disease (0.4), respiratory illnesses (2.3%), hypertension (6.6%). HIV-positive status was reported by 126 (12.4%) participants. The study participants were urban residents with a high proportion having access to electricity (87.7%), refrigerator (75.8%) and television (88%) ownership. Having a ‘battery or generator for power’ was reported by 240 (23.6%) of participants. Ownership of these resources was used to classify participants as high, middle, or low socioeconomic status (Table 1). Among study participants, 428 (42.1%) reported knowing someone who was seriously ill or died from COVID-19 disease; these were close relatives (e.g., siblings, spouse or parents) in 30% of respondents. Internet use in the past 30 days was reported by 420 (41.4%) participants (Table 1).

### 3.1. Vaccine Convenience

Time to travel to the vaccination site was <15 min for 443 (43.6%) participants. Time taken to receive the shot after arrival was less than 10 min for 338 (33.3%) and less than an hour for 839 (82.6%). Appointments were not needed to receive the vaccine. Finding a vaccination site was described as ‘very easy’ by 853 (84.2%), getting to the vaccination center was ‘very easy’ for 889 (87.7%), financially affording transportation to the site was ‘very easy’ for 877 (86.5%) and finding a vaccination site with convenient hours was ‘very easy’ for 786 (77.5%) participants. When asked about barriers to accessing vaccination, 106 (10.5%) participants indicated that they had to arrange for childcare, 207 (20.4%) had to take time off from paid work and 112 (11%) earned less money because they would have to take time from work. 

### 3.2. Vaccine Confidence

When asked “When the COVID-19 vaccines first became available, what was your view towards COVID-19 vaccines?”, 477(47%) had ‘very negative views’ and 198 (19.5%) had ‘somewhat negative’ views. Women were more likely than men (OR 1.51 (95% CI: 1.16, 1.97, *p* = 0.002)) and young adults were more likely than youth (OR 1.37 (95%CI: 1.01, 1.86, *p* = 0.043)) to have ‘very negative’ or ‘somewhat negative’ views towards COVID-19 vaccines (Table 2). 

A third of participants (32.6%) had major concerns about receiving the COVID-19 vaccine. Gender, age, and HIV status were associated with differences in major concerns. Women were more likely than men (OR 1.45 (95%CI: 1.1, 1.92), *p* = 0.009) to have major concerns (Table 3). Young adults (OR 1.44 (95% CI: 1.04, 2.00, *p* = 0.030) and older adults (OR 2.33 (95%CI: 1.59, 3.42, *p* < 0.001) were more likely than youth to have major concerns (Table 3). Individuals who knew someone who became seriously ill or died because of COVID-19 were less likely to have negative views (OR 0.69 (95%CI: 0.53–0.91, *p* = 0.007)) or major concerns (OR 0.71 (95%CI: 0.54–0.94, *p* = 0.017)) compared to those who did not (Table 2 and Table 3).

Immediate health concerns were a concern for 602 (59.3%) and long-term health effects were a concern for 520, (51.2%). Young adults compared to youth (OR 1.48 (95%CI: 1.11, 1.98), *p* = 0.008) and PLWH (OR 1.56 (95%CI: 1.05–2.23, *p* = 0.029) compared to those without were more likely to express concerns about immediate side effects (Table 3). Long-term health effects were of particular concern for women compared to men, and young adults compared with youth (Table 3). Those with personal knowledge of someone who was seriously ill or died from COVID-19 were less likely to indicate concerns about long-term health effects (OR 0.69 (95% CI: 0.54, 0.88, *p* = 0.003) (Table 3). 

The concern that ‘the vaccine has not been tested enough’ was expressed by 223 (21.9%), that the ‘vaccine was still new’ by 190 (18.7%) and that there was no ‘need for a vaccine’ was expressed by 167 (16.4%). Among the respondents, 293 (28.8%) chose ‘other’ for their concerns. The 293 participants’ concerns focused on fear of death, infertility, health effects of vaccination, interaction with medications and comorbid conditions, and interference with pregnancy and breastfeeding, as well as conspiracy theories (Table 4). 

Despite concerns about immediate and long-term effects of the vaccines, 728 (71.5%) participants strongly agreed with the statement “In general COVID-19 vaccines are safe”. Participants with lower educational attainment and without access to the Internet were more likely to strongly agree that vaccines are safe (Supplementary Table S1). PLWH had higher odds of perceiving vaccines as safe compared to those without HIV (OR 1.71 (95%CI: 1.07, 2.74, *p* = 0.025) (Table S1). Although most respondents felt that the Sinovac/Sinopharm vaccine was either very safe (500 (49.2%)) or somewhat safe (175 (17.2%)), 311 (30.6%) indicated that they did not know if the vaccine that they had received was safe. 

Perceived vaccine effectiveness was high, with 699 (68.8%) participants strongly agreeing with the statement “I am confident that COVID-19 vaccines are effective in preventing the disease”. The odds that one perceived COVID-19 vaccines to be effective in preventing disease decreased with increasing education and with Internet use (Table S2). PLWH compared to those without HIV (OR 1.68 (95%CI: 1.07, 2.64) and those with a personal experience with COVID-19 compared to those without (OR 1.47 (95%CI: 1.12, 1.95, *p* = 0.006) had higher odds to strongly agree that COVID-19 vaccines were effective in preventing disease (Table S2).

Perceived confidence in national regulatory processes was high, with 728 (71.7%) strongly agreeing with the statement “I am confident that my country’s regulatory process approved the COVID-19 vaccine only when it was shown to be safe”. Increasing levels of education and Internet use were associated with lower odds of strongly agreeing with the statement (Table S3). Participants with a personal experience with COVID-19 (OR 1.37 (95%CI: 1.03, 1.82), *p* = 0.028) were more likely to be confident in national regulatory processes. Similarly, PLWH were more likely than those without HIV (OR 1.79 (95%CI: 1.11, 2.89), *p* = 0.017) to strongly agree with the statement (Table S3).

Internet users consistently had lower perceived confidence in vaccine safety (OR 0.72 (95%CI: 0.55, 0.95), *p* = 0.02), lower perceived confidence in vaccine effectiveness (OR 0.61 (95%CI: 0.47, 0.50, *p* < 0.001) and lower perceived confidence in regulatory processes (OR 0.64 (95%CI: 0.48, 0.85, *p* = 0.002) than those that had not used the Internet in the last 30-days (Tables S1–S3). 

### 3.3. Key Influencers

Normative behavior can be a key motivator of vaccine uptake. The influence of others was important with 684 (67.3%) participants indicating that they strongly agreed with the statement “I came to get vaccinated because people important to me encouraged me to be vaccinated”. In addition, 702 (69.1%) participants strongly agreed with the statement “I came to get vaccinated today because people important to me got a COVID-19 vaccine”. Family members were key influencers in getting vaccinated for 561 (55.2%) participants. Religious leaders (98, (9.7%)), community leaders (56, (5.5%)), healthcare workers (133, (13.1%)) and co-workers (137 (13.5%)) were less commonly listed as key influencers.

**Figure 1:**
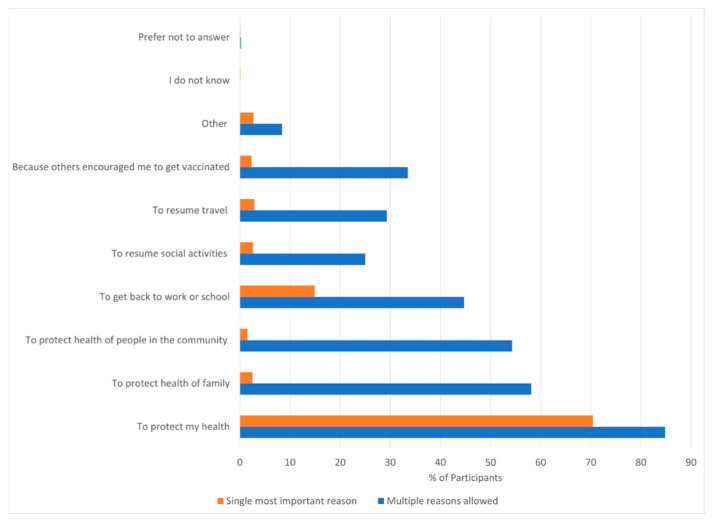
Reasons for presenting for vaccination today among late adopters of vaccination. Participants were asked “Why did you get vaccinated today?”. Multiple responses were allowed (green). The subsequent follow up question was “What is the most important reason you got vaccinate today?”, a single response was allowed.

The reason for getting vaccinated today was to ‘protect my health’ for 862 (84.8%), to ‘protect the health of my family’ for 590 (58.1%), ‘to protect health of the people in the community’ for 552 (54.3%) and ‘to get back to work or school’ for 451 (44.7%) (Supplementary Figure S7). When asked the single most important reason for getting vaccinated today the top two most important reasons were ‘To protect my health’ for 716 (70.4%) participants, and to ‘get back to work or school’ for 152 (14.9%) (Figure 1). Among PLWH, protection of the health of the family (68.3% vs. 56.6%, *p* < 0.001), and the health of the community (65.1% vs. 52.8%, *p* < 0.001) were more frequently cited as reasons for vaccination compared to those without HIV infection. Resumption of social activities (2.6%), travel (2.9%) and ‘because others encouraged me to’ (2.3%) were the single most important reasons for a very small number of participants (Figure 1). 

### 3.4. Sources of Information

**Figure 2:**
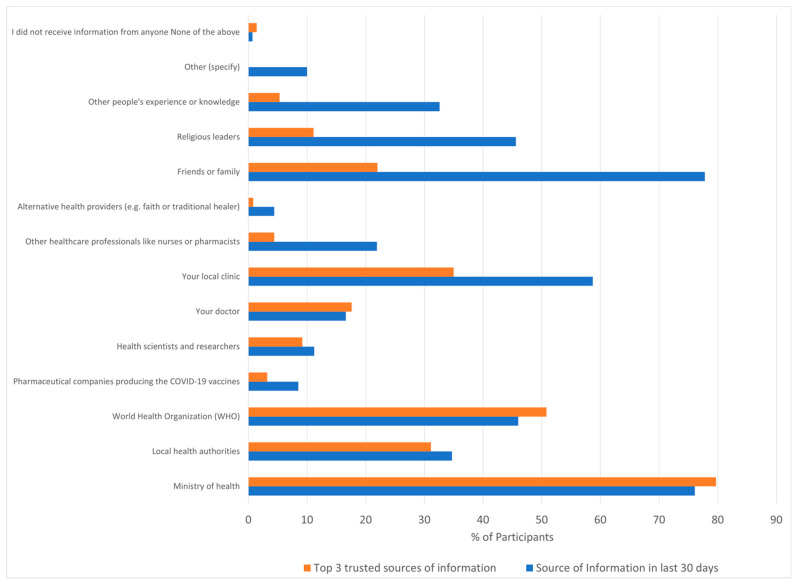
Sources and Trusted sources of information. Participants were asked “In the past 30 days, who have you received information from about COVID-19?” multiple responses were allowed (Green). Participants were subsequently asked “What are your top three sources of trusted information about COVID-19 vaccines?” (Red—only three responses allowed).

We asked the question ‘In the past 30 days, who have you received information from about COVID-19?’. Friends or family were the most common sources of information for 790, (77.8%), Ministry of Health for 773 (76.1%), local clinic for 596 (58.7%), religious leaders for 463 (45.6%), and the World Health Organization (WHO) for 467 (46%) (Figure 2). When asked about the top three sources of trusted information about COVID-19 vaccines, the primary sources were the Ministry of Health (79.7%), WHO (50.8%), local clinic (35%), local health authorities (31.1%), and friends or family (22%) (Figure 2). The WHO was a more important source of information for men than women (50.4% vs. 41.5%, *p* < 0.05), whereas women more frequently listed their local clinic (52.2% vs. 65.2%, *p* < 0.0001) and religious leaders (56.3% and 34.8%, *p* < 0.0001) among their top three trusted sources for information. Doctors and other health professions were in the top three trusted sources for 16.6% and 21.9% respectively, and alternative health providers for 4.4%. 

We assessed the use of media as an information source by asking ‘In the past 30 days, where have you obtained information about COVID-19 vaccines?’. The radio (86.5%), television (76.7%), WhatsApp (62.2%) and Facebook (36.9%) were the primary media sources of information. Radio use had high penetration and was more likely to be a source for young adults compared with youth (OR 1.85 (95%CI 1.2–2.84), *p* = 0.005) (Table S4). Although television had high penetration as an information source, its use tracked with education. The odds of receiving information from television were twice as high in those with lower secondary (OR 2.22 (95%CI 1.39–3.56), *p* = 0.001) and higher secondary education (OR 2.72 (95%CI 1.35–5.48), *p* = 0.005) than those with primary education only (Table S5). Social media, primarily WhatsApp and Facebook, as a source of information tracked strongly with age and education. Older adults had much lower odds of obtaining information from WhatsApp (OR 0.41 (95% CI 0.3–0.58), *p* < 0.001) (Table S6) or Facebook (OR 0.3 (95% CI (0.2–0.45), *p* < 0.001) compared to youth (Table S7), and similarly, for those with secondary and tertiary education compared to those with primary education (Table S6). 

## 4. Discussion

The WHO has set an ambitious goal for COVID-19 vaccination anticipating that over 70% of populations globally will be vaccinated by the end of 2022 [14]. However, vaccine hesitancy is threatening that goal. Vaccine hesitancy is defined by convenience, confidence, and complacency towards vaccines. Removing practical barriers to vaccination has strengthened various vaccination programs [19,20,21]. Improving access through community based on mobile vaccination access points can significantly improve vaccination uptake, particularly among marginalized populations [22]. In this study of late adopters in an urban setting, convenience was no longer major a barrier with most participants indicating that the sites were accessible and the service efficient with relatively low waiting times. However, up to a fifth indicated concerns about lost income and taking time off as a barrier to vaccination. Improving access points, including mobile vaccination centers at both formal places of employment and informal places of employment, may improve vaccine uptake rates particularly in settings of low levels of formal employment such as urban Zimbabwe [23].

Vaccine confidence based on perceptions of safety, effectiveness and trust in regulatory processes can influence vaccine uptake. Perceptions of COVID-19 vaccines when they first came out were largely negative among late adopters, likely contributing to them taking a ‘wait and see’ approach. In our study of late adopters, female gender and increasing age were more likely to be associated with negative perceptions. A study conducted in Botswana prior to widespread vaccine availability found that males had higher odds of accepting vaccines compared with females [24]. Similar trends of higher female vaccine hesitancy have also been observed in Australia [25]. These gender differences are contributing to gender inequities in vaccine uptake higher levels of hesitancy among women [26,27,28]. This could have significant implications in general vaccine uptake, as women are often the primary decision makers with regards to family health. Women in this cohort had concerns about vaccine safety. Information on vaccine safety in general has not been effectively communicated and up to a third of participants indicated that they did not know if the vaccine that they received was safe. Communication strategies to enhance vaccine coverage will need to be gender specific and focused on information around the safety of specific vaccines. The lack of consistent communication on vaccine safety in pregnancy, and ongoing social media misinformation about fertility may be important drivers of delayed vaccine uptake among women. Women’s safety concerns were focused on the effects on pregnancy, breastfeeding and fertility [29,30].

Enhancing COVID-19 vaccination uptake among PLWH especially in countries such as Zimbabwe with high HIV seroprevalence [31] will be important. People living with HIV, particularly those that are viremic or have low CD4 counts, may be at increased risk of hospitalization and death from COVID-19 [32,33]. In addition, immunocompromised individuals, including people living with uncontrolled HIV infection may be an important source for ongoing SARS-CoV-2 evolution [34]. Within this study cohort, PLWH were almost twice as likely to have major concerns about COVID-19 vaccines and side effects than those without HIV. They were concerned about the impact on their health as well as interactions with their antiretroviral therapy (ART). Vaccination rates among PLWH across different geographies are not fully understood [35,36]. However, vaccination coverage targets for PLWH will likely need to be higher than those for the non-immunocompromised population. This is particularly important in settings where a large proportion of PLWH are not yet on ART or have advanced HIV disease [37]. This group of individuals may be ideal for focused vaccination campaigns, as results from this study suggest that PLWH were more likely to perceive vaccines as safe and effective and trust government regulatory processes. A survey among PLWH in Uganda similarly found high levels of trust in government officials and significant willingness for COVID-19 vaccines [38]. This is likely because PLWH on chronic therapy interact with the public healthcare system often and have come to trust in and rely on the services. 

Perceptions of safety, effectiveness and trust in regulatory authorities also tracked significantly with education and income status. Several studies have evaluated the correlation between education level and vaccine hesitancy with significant differences across cultures and populations. In some setting higher levels of education correlate with vaccine uptake while in others it does not [39,40,41]. In this study, participants with higher education and better resources were less likely to perceive the vaccines as effective or trust in regulatory processes. This may be an important trend to watch closely as the more educated and higher income individuals are often key influencers in Zimbabwean families and communities. Communication strategies that effectively target these socio-economic groups will be important, particularly as their access of social media-based information and disinformation is higher. 

Vaccine distribution logistics and access are heavily dependent on health systems infrastructure and capabilities. However actual uptake of adult and adolescent vaccines is heavily influenced by personal beliefs that are amenable to change through inter-personal influence [42,43]. Social influence was shown to have a role in uptake of COVID-19 vaccines and prevention measures among adults and adolescents in western settings [44,45,46]. In this study, social influence was a key factor in motivating vaccine uptake. Descriptive norms i.e., beliefs about what others are doing regarding the behavior were important motivators. The encouragement of others and the vaccination behaviors of ‘people important to them’ influenced this cohort of late adopters to seek vaccination. Family members were the most important influencers.

The study explored the reasons for vaccination. Protecting one’s own health was a major driver of vaccination in over 80% of late adopters. Protecting the health of the family and the community were important to more than half of the late adopters. These attitudes may be driven by social norms, as well as altruism. In western cohorts, altruistic reasoning has been shown to be an important factor in vaccine uptake decision-making [47]. In this study, we show that similar attitudes play an important role in vaccine uptake among African late adopters. 

Risk perception is an important driver of uptake of health interventions. Having a close personal connection with a disease and its severity can influence decision-making. A study of pediatric influenza immunization in Kenya showed that sibling experience with disease influenced vaccine uptake [10]. Similarly, personal experience in knowing someone who was seriously ill or died from COVID-19 had a significant impact on attitudes towards vaccines. This prior personal experience likely influenced risk perception driving vaccination uptake among late adopters. Other key influencers were religious leaders and local clinics particularly for women. Local nurse-led clinics are generally free in Zimbabwe and more accessible than other clinics with other health providers such as medical doctors [48]. Strengthening the capacity for religious leaders and local community-based nurses to provide important information on vaccine safety and effectiveness will be important for improving vaccine uptake. 

This pandemic has shown the importance of media and communications in guiding vaccine uptake. Social media and the anti-vax movement are global and have become an important driver of vaccine hesitancy [30,49]. Politics and trust in governments have been important in driving vaccine uptake in various parts of the world [50,51,52]. Although levels of trust in government may be low in many countries, we found in this study that trust in government as a source of information on health and specifically COVID-19 was very high. Most participants cited the Ministry of Health as a top trusted source of information. Building and maintaining that trust in Health Ministries and regulatory processes will be critical in ensuring the success of COVID-19 and other vaccination programs. 

Traditional media—radio and television—were the main media sources of information. Internet-based media sources were less commonly cited as major sources with only 41% of the cohort using the Internet over the last 30 days. Television and radio are largely state controlled, providing the authorities with an opportunity to disseminate accurate information on the safety and effectiveness of vaccines. However, social media, particularly WhatsApp, is growing in importance and as a source of information among the youth, and those with a higher economic status and more education. Communication strategies to enhance vaccine uptake should use both traditional and social media platforms but must be targeted by gender and age to be most effective.

The study has limitations, some based on geography and timing. The study was conducted in a large city and focused on an urban population at a time when access had improved significantly. Vaccination had become widely distributed in community clinics managed by the City of Harare. We enrolled participants from some of the largest and busiest vaccination clinics that were community based as well as outreach sites. The study, however, does not reflect the access and convenience for those throughout Zimbabwe particularly in rural settings. Despite this limitation, the study design enabled us to evaluate late adopters in a setting where some access barriers had been minimized, providing insight into other environmental and individual factors that may influence vaccine uptake. Further understanding of barriers, motivations, and attitudes among rural populations in Zimbabwe and other African countries will be important. 

An important limitation is that our survey was not designed to specifically evaluate the role of complacency in-depth. We observed that as many as 16.4% of participants indicated that they ‘Do not see need for vaccine’. Complacency has been described as a major barrier in Africa and may become increasingly important as milder variants of SARS-CoV-2 emerge and seroprevalence from natural infection increases [53]. 

The study provides insights into late adopters of vaccination in Zimbabwe one year after the national COVID-19 vaccination program began and offers insights into vaccine hesitant individuals who may be open to vaccination. Because we studied those that made the decision to be vaccinated, this research does not provide insights into those that have chosen not to get a vaccine, or those who remain undecided. We conducted focus group research in cohorts of individuals who are not yet vaccinated to further define their attitudes motivations and barriers. The nature of this study was to focus on those presenting for vaccination to obtain actionable insights from a large cohort. Ongoing analysis of qualitative data from focus groups will provide further insights into the attitudes of those that remain unvaccinated.

Healthcare workers can be important drivers of vaccine hesitancy in the community. This may be driven by their own perceptions and objective knowledge of vaccine safety and efficacy. The study is limited in that we did not assess the attitudes to COVID-19 vaccines of healthcare workers. In sharing this data with healthcare workers, many indicated that their own personal knowledge about vaccines was limited, and they would have appreciated further training and vaccine knowledge reinforcement. The impact of healthcare worker hesitancy on program success is critical and needs to be studied and addressed [54,55].

The strengths of the study are that it provides key insights into vaccine hesitancy in a large urban African setting. The survey evaluated participants at static as well as outreach sites, and with broad coverage across Harare. In addition, the study had a representative proportion of people with key morbidities such as HIV infection. The focus on late adopters enabled the study to identify the key focus areas for an effective COVID-19 vaccine uptake promotion strategy. The study data highlights the need for consistent and targeted information that is gender and age specific and targeted to subpopulations such as PLWH. Information must be delivered by trusted sources, such as the Ministry of Health, via traditional media platforms, such as radio and television, that are heavily accessed and can be used to address misinformation delivered on social media. The vaccination encounter should equip friends and family with accurate information on specific vaccines and side effects so they can act as informed social influencers for their family and friends. These individuals can be key ambassadors for vaccination within their social network. In addition, educating religious leaders who have influence over their congregations and are key sources of information, particularly for women, will be important. Vaccine hesitancy can only be tackled effectively by taking on a targeted approach to health communications that builds trust within communities using trusted communicators. 

## 5. Conclusions

Late adopters were motivated to seek vaccination in order to protect their own health and the health of the family and community. Late adopters are concerned about both immediate and long-term health effects of COVID-19 vaccination. Late adopters are heavily influenced by the attitudes and behaviors of others towards vaccination, particularly friends and family. The need for more information on vaccine safety was evident, and trusted sources included the Ministry of Health, friends and family, WHO, and local clinics with significant use of traditional media (radio and television) to access information. Age, gender, HIV status, and knowing someone who had COVID-19, were associated with differences in attitudes to vaccination. Insights from this study highlight the importance of normative behavior in adult vaccination decision-making and suggest that tailor-made messages that demystify vaccination will be critical to convert those taking a ‘wait and see’ approach into late adopters. 

## Figures and Tables

**Table 1:** Baseline cohort demographics.

Characteristic	N (%)
Gender (Female)	508 (50%)
Median age (IQR)	30 (22–39)
Age groups	
18–25 (Youth)	368 (36.2%)
26–39 (Young adults)	409 (40.3%)
≥40 (Older adults)	239 (23.5%)
Ethnicity (Black African)	1016 (100%)
Highest Level of Education	
No formal schooling	5 (0.5%)
Primary school ^1^	84 (8.3%)
Lower Secondary school ^2^	735 (72.4%)
Higher Secondary ^3^	117 (11.5%)
Tertiary Education	74 (7.3%)
Co-morbid conditions	
Diabetes	15 (1.5%)
Cardiac Disease	4 (0.4%)
Respiratory Illness	23 (2.3%)
Hypertension	67 (6.6%)
HIV	126 (12.4%)
Socioeconomic status	
High ^4^	172 (16.9%)
Middle ^5^	598 (58.9%)
Low ^6^	246 (24.2%)
Internet use	
In the past 30 days have you used the Internet?	420 (41.4%)
Personal COVID experience	
Know someone who became seriously ill or died as a result of COVID	428 (42.1%)

^1^ Primary up to 7th grade. ^2^ High school completing up to and including Ordinary Level (GSCE or 11th Grade). ^3^ Higher secondary completing Advanced Level (or 13th Grade). ^4^ High socioeconomic status defined by owning a generator or battery as an alternate source of power and a refrigerator. ^5^ Middle socioeconomic status owned one but not the other. ^6^ Low socioeconomic status neither owned a refrigerator or a generator.

**Table 2:** Factors associated with having a very negative or somewhat negative view towards COVID-19 vaccines when they first became available.

			Univariate		Multivariate	
Variable	Category	Frequency (N, %)	OR (95% CI)	*p*-Value	OR (95% CI)	*p*-Value
Gender	Male	508 (50.0%)	1			
	Female	508 (50.0%)	1.573 (1.209, 2.045)	0.001	1.508 (1.156,1.967)	0.002
Age (y)	18–25	368 (36.2%)	1			
	26–39	409 (40.3%)	1.446 (1.069, 1.957)	0.017	1.371 (1.01, 1.862)	0.043
	≥40	239 (23.5%)	0.931 (0.665, 1.304)	0.679	0.869 (0.617, 1.223)	0.42
Education	Primary *	89 (8.8%)	1			
	Lower secondary	735 (72.4%)	1.179 (0.747, 1.861)	0.481		
	Higher Secondary	117 (11.5%)	1.326 (0.741, 2.374)	0.342		
	Tertiary	74 (7.3%)	1.026 (0.541, 1.945)	0.938		
Economic status	High	172 (16.9%)	1			
	Middle	598 (58.9%)	0.885 (0.618, 1.270)	0.508		
	Low	246 (24.2%)	1.122 (0.738, 1.707)	0.590		
Personal COVID Experience	No	428 (42.1%)	1			
	Yes	588 (57.9%)	0.669 (0.514, 0.870)	0.003	0.693 (0.53, 0.905)	0.007
HIV status	Negative	126 (12.4%)	1			
	Positive	890 (87.6%)	1.054 (0.708, 1.569)	0.795		
Internet use in last 30 days	No	420 (41.4%)	1			
	Yes	594 (58.6%)	0.773 (0.594, 1.006)	0.056		

* Primary up to 7th grade, Lower secondary: High school completing up to and including Ordinary Level (GSCE or 11th Grade); Higher secondary completing Advanced Level (or 13th Grade).

**Table 3:** Major concerns about COVID-19 vaccine, Immediate side effects and Long-term health effects.

		Major Concerns		Immediate Side Effects		Long-Term Health Effect	
Variable	Category	OR (95% CI)	*p*-Value	OR (95% CI)	*p*-Value	OR (95% CI)	*p*-Value
Gender	Male	1		1		1	
	Female	1.454 (1.098, 1.923)	0.009 *	1.342 (1.044, 1.725)	0.022 *	1.416 (1.106, 1.812)	0.006 *
Age (years)	18–25	1		1		1	
	26–39	1.439 (1.036, 1.998)	0.030 *	1.48 (1.109, 1.975)	0.008 *	1.608 (1.211, 2.135)	0.001 *
	≥40	2.329 (1. 585, 3.422)	<0.001 *	1.055 (0.76, 1.465)	0.749	1.142 (0.824, 1.582)	0.426
Education	Primary	1		1		1	
	Lower secondary	0.938 (0.576, 1.526)	0.796	0.949 (0.603, 1.491)	0.819	1.172 (0.754, 1.821)	0.48
	Higher Secondary	1.162 (0.614, 2.199)	0.645	0.773 (0.441, 1.355)	0.369	1.219 (0.702, 2.117)	0.482
	Tertiary	1.338 (0.665, 2.688)	0.414	0.586 (0.313, 1.094)	0.093	1.317(0.709, 2.443)	0.383
Economic status	High	1		1		1	
	Middle	0.842 (0.577, 1.228)	0.371	1.075 (0.709, 1.560)	0.803	1.28 (0.866, 1.891)	0.215
	Low	1.142 (0.733, 1.779)	0.558	0.905 (0.642, 1.278)	0.572	1.016 (0.723, 1.427)	0.926
Personal Covid Experience	No	1		1		1	
	Yes	0.710 (0.536, 0.940)	0.017 *	0.852 (0.661, 1.097)	0.215	0.688 (0.536, 0.884)	0.003 *
HIV status	Negative	1		1		1	
	Positive	1. 250 (0.83, 1.884)	0.285	1.558 (1.047, 2.23)	0.029 *	1.527 (1.044, 2.233)	0.029 *
Internet use in last 30 days	No	1		1		1	
	Yes	1.210 (0.896, 1.635)	0.214	0.778 (0.604, 1.003)	0.053	1.158 (0.902, 1.488)	0.25

* Significant *p* value (*p* < 0.05).

**Table 4:** Summary of the concerns expressed by study participants who listed concerns as “other”.

Concern	Frequency (N = 293)	Example Statements and Thematic Areas Regarding “Other Concerns”
Death	75 (25.6%)	Feared from hearsay that vaccines would kill after a certain time Feared death and becoming a “Zombie” after vaccination Feared dying soon after vaccination I am afraid that I will not survive for 2 years after receiving the vaccine
Health Effect of vaccination	43 (14.68%)	“Someone in Bulawayo got vaccinated and experienced necrosis” Feared fainting or stroke due to vaccination, too many people at site Husband was saying no-one in his house gets vaccinated because he has family members who “got sick” after vaccination Feared that vaccine would distort body parts Blindness Blood clotting
Prevailing conspiracy theories	27 (9.22%)	Feared vaccine was to depopulate Some doctors from affected countries spoke negatively about vaccines. It is a created disease to wipe out people, dosage for Africans may be deadly Feared some foreign agent instead of the vaccine being injected into him “I think the whites want to depopulate Africans”
Pregnant or Breastfeeding concerns/interactions	27 (9.22%)	Was pregnant so feared for baby I was pregnant when it started so was afraid to affect baby Effects of vaccine on pregnant wife Breast milk may dry off I was pregnant so was told l can’t
Lack of trust or understanding of vaccine/manufacturer	26 (8.87%)	Was not trusting the vaccine Feared the coronavirus being injected into him instead of actual vaccine Concerned about manufacturers of the vaccine, that it came from China Feared that the vaccine was fake
Effect on Fertility	21 (7.17%)	The vaccine causes infertility
Drug-Drug interactions and/or comorbid conditions	21 (7.17%)	Feared negative interactions between vaccine and underlying diabetes issue Won’t it affect my BP? Feared contraindications between TB medication he was taking and vaccine
Injection site pain/swelling, fear of needles	16 (5.46%)	Fear of needles
Convenience of vaccination	11 (3.75%)	“Social media was saying bad things so we were afraid to come, it’s also taking too long in the queue, 1 nurse dealing with too many people” The vaccine site a bit far from home Identification documents were not close to him, so he couldn’t get vaccinated
HIV infection and/or co-interaction with ART	10 (3.41%)	Fear since I am HIV positive Did not understand the whole vaccination issue, was afraid of vaccination during ART
Fear	7 (2.39%)	Was afraid of the COVID 19 test that is done before vacation;
Worse COVID disease and concern of virus in vaccine	6 (2.05%)	You get COVID Feared being injected by the virus itself whilst they pose it as a “vaccine” Feared the coronavirus being injected into him instead of actual vaccine
No need for vaccine/Did not want vaccine	2 (0.68%)	
Social impact	1 (0.34%)	Fear that people will gossip that am vaccinated

## Data Availability

The data presented in this publication will be openly available in a repository with DOI number at the time of publication.

## Supplementary Materials

The following supporting information can be downloaded at: https://www.mdpi.com/article/10.3390/vaccines11020411/s1, Table S1. Perceived confidence in safety of COVID-19 vaccines. Participants indicating that they strongly agree with the statement ‘In general, COVID-19 vaccines are safe’. Table S2. Perceived confidence in vaccine effectiveness. Participants responding that they strongly agree with the statement ‘I am confident that COVID-19 vaccines are effective in preventing the disease’. Table S3. Perceived government trust. Participants indicating that they strongly agree with the statement ‘I am confident that my country’s regulation process approved the COVID-19 vaccine, only when it was shown to be safe’. Table S4. Primary sources of media information: Radio. Table S5. Primary sources of media information: Television. Table S6. Primary sources of media information: Whatsapp. Table S7. Primary sources of media information: Facebook.
