# Supplementary material for: Investigating Attitudes, Motivations and Key Influencers for COVID-19 Vaccination Uptake among Late Adopters in Urban Zimbabwe
Source: Vaccines (Basel). 2023 Feb 10;11(2):411. doi: 10.3390/vaccines11020411 (PMC9958877; doi:10.3390/vaccines11020411)
Supplement: Supplementary file 1 [file vaccines-11-00411-s001.zip › vaccines-2102702-supplementary.pdf]

Table S1. Perceived confidence in safety of COVID-19 vaccines. Participants indicating that they strongly agree with the statement 'In general, COVID-19 vaccines are safe'.

Safety						
**Variable**	Category	Frequency	Univariate		Multivariate	
			OR (95% CI)	p-value	OR (95% CI)	p-value
**Gender**	Male	508 (50.0%)	1			
	Female	508 (50.0%)	1.101 (0.839, 1.446)	0.487		
**Age**	18-25	368 (36.2%)	1			
	26-39	409 (40.3%)	1.16 (0.85, 1.583)	0.349		
	≥40	239 (23.5%)	1.147 (0.8, 1.644)	0.456		
**Education**	Primary*	89 (8.8%)	1			
	Lower Secondary	735 (72.4%)	0.503 (0.278, 0.91)	0.023		
	Higher Secondary	117 (11.5%)	0.404 (0.202, 0.805)	0.01		
	Tertiary	74 (7.3%)	0.159 (0.076, 0.33)	<0.001		
**Economic Status**	High	172 (16.9%)	1			
	Middle	598 (58.9%)	1.188 (0.827, 1.708)	0.351		
	Low	246 (24.2%)	1.643 (1.065, 2.537)	0.025		
**Personal Covid Experience**	No	428 (42.1%)	1			
	Yes	588 (57.9%)	1.275 (0.965, 1.686)	0.087		
**HIV**	Negative	126 (12.4%)	1			
	Positive	890 (87.6%)	1.812 (1.135, 2.891)	0.013	1.71 (1.068, 2.738)	0.025
**Internet use in the last 30 days**	No	420 (41.4%)	1			
	Yes	594 (58.6%)	0.697 (0.529, 0.917)	0.01	0.722 (0.548, 0.953)	0.021

Table S2. Perceived confidence in vaccine effectiveness. Participants responding that they strongly agree with the statement 'I am confident that COVID-19 vaccines are effective in preventing the disease'.

			Effectiveness			
**Variable**	Category	Frequency	Univariate		Multivariate	
			OR (95% CI)	p-value	OR (95% CI)	p-value
**Gender**	Male	508 (50.0%)	1			
	Female	508 (50.0%)	1.213 (0.930, 1.582)	0.155		
**Age**	18-25	368 (36.2%)	1			
	26-39	409 (40.3%)	1.429 (1.057, 1.933)	0.02		
	≥40	239 (23.5%)	1.501 (1.053, 2.139)	0.025		
**Education**	Primary*	89 (8.8%)	1			
	Lower Secondary	735 (72.4%)	0.503 (0.283, 0.897)	0.02		
	Higher Secondary	117 (11.5%)	0.291 (0.15, 0.567)	<0.001		
	Tertiary	74 (7.3%)	0.146 (0.071, 0.301)	<0.001		
**Economic Status**	High	172 (16.9%)	1			
	Middle	598 (58.9%)	1.952 (1.286, 2.961)	0.002		
	Low	246 (24.2%)	1.556 (1.096, 2.209)	0.013		
**Personal Covid Experience**	No	428 (42.1%)	1			
	Yes	588 (57.9%)	1.481 (1.126, 1.948)	0.005	1.474 (1.118, 1.945)	0.006
**HIV**	Negative	126 (12.4%)	1			
	Positive	890 (87.6%)	1.772 (1.132, 2.774)	0.012	1.677 (1.065, 2.64)	0.026
**Internet use in the last 30 days**	No	420 (41.4%)	1			
	Yes	594 (58.6%)	0.584 (0.446, 0.764)	<0.001	0.613 (0.467, 0.804)	<0.001

Table S3. Perceived government trust. Participants indicating that they strongly agree with the statement 'I am confident that my country's regulation process approved the COVID-19 vaccine, only when it was shown to be safe.'

			Trust in Regulatory			
**Variable**	Category	Frequency	Univariate		Multivariate	
			OR (95% CI)	p-value	OR (95% CI)	p-value
**Gender**	Male	508 (50.0%)	1			
	Female	508 (50.0%)	1.168 (0.889, 1.535)	0.266		
**Age**	18-25	368 (36.2%)	1			
	26-39	409 (40.3%)	1.418 (1040, 1.935)	0.027		
	≥40	239 (23.5%)	1.399 (0.974, 2.008)	0.069		
**Education**	Primary	89 (8.8%)	1			
	Lower Secondary	735 (72.4%)	0.932 (0.555, 1.562)	0.788		
	Higher Secondary	117 (11.5%)	0.551 (0.297, 1.023)	0.059		
	Tertiary	74 (7.3%)	0.235 (0.12, 0.461)	<0.001		
**Economic Status**	High	172 (16.9%)	1			
	Middle	598 (58.9%)	1.307 (0.913, 1.87)	0.143		
	Low	246 (24.2%)	2.154 (1.387, 3.346)	0.001		
**Personal Covid Experience**	No	428 (42.1%)	1			
	Yes	588 (57.9%)	1.39 (1.049, 1.841)	0.022	1.374 (1.034, 1.825)	0.028
**HIV**	Negative	126 (12.4%)	1			
	Positive	890 (87.6%)	1.899 (1.182, 3.051)	0.008	1.79 (1.108, 2.891)	0.017
**Internet use in the last 30 days**	No	420 (41.4%)	1			
	Yes	594 (58.6%)	0.608 (0.461, 0.801)	<0.001	0.639 (0.484, 0.845)	0.002

Table S4. Primary sources of media information: Radio

Radio						
**Variable**	Category	Frequency	Univariate		Multivariate	
			OR (95% CI)	p-value	OR (95% CI)	p-value
**Gender**	Male	508 (50.0%)	1			
	Female	508 (50.0%)	0.951 (0.663, 0.363)	0.783		
**Age**	18-25	368 (36.2%)	1			
	26-39	409 (40.3%)	1.783 (1.167, 2.725)	0.007	1.846 (1.199, 2.843)	0.005
	≥40	239 (23.5%)	1.158 (0.737, 1.819)	0.524	1.292(0.795, 2.1)	0.301
**Education**	Primary	89 (8.8%)	1			
	Lower Secondary	735 (72.4%)	2.254 (1.29, 3.939)	0.004	2.318(1.29, 4.164)	0.005
	Higher Secondary	117 (11.5%)	1.493 (0.731, 3.048)	0.271	1.624(0.762, 3.458)	0.209
	Tertiary	74 (7.3%)	0.685 (0.335, 1.402)	0.3	0.685(0.326, 1.443)	0.32
**Economic Status**	High	172 (16.9%)	1			
	Middle	598 (58.9%)	1.241 (0.772, 1.996)	0.372		
	Low	246 (24.2%)	1.245 (0.716, 2.167)	0.438		
**Personal Covid Experience**	No	428 (42.1%)	1			
	Yes	588 (57.9%)	1.179 (0.815, 1.705)	0.381		
**HIV**	Negative	126 (12.4%)	1			
	Positive	890 (87.6%)	1.176 (0.663, 2.083)	0.58		
**Internet use in the last 30 days**	No	420 (41.4%)	1			
	Yes	594 (58.6%)	1.101 (0.762, 1.591)	0.609		

Table S5. Primary sources of media information: Television

Television						
**Variable**	Category	Frequency	Univariate		Multivariate	
			OR (95% CI)	p-value	OR (95% CI)	p-value
**Gender**	Male	508 (50.0%)	1			
	Female	508 (50.0%)	0.811 (0.606, 1.086)	0.159		
**Age**	18-25	368 (36.2%)	1			
	26-39	409 (40.3%)	1.008 (0.722, 1.406)	0.963		
	≥40	239 (23.5%)	0.997 (0.678, 1.464)	0.986		
**Education**	Primary	89 (8.8%)	1			
	Lower Secondary	735 (72.4%)	2.458 (1.558, 3.877)	<0.001	2.222 (1.386, 3.56)	0.001
	Higher Secondary	117 (11.5%)	4.186 (2.153, 8.138)	<0.001	2.719 (1.35, 5.475)	0.005
	Tertiary	74 (7.3%)	2.214 (1.124, 4.361)	0.022	1.203 (0.578, 2.505)	0.621
**Economic Status**	High	172 (16.9%)	1			
	Middle	598 (58.9%)	0.734 (0.465, 1.159)	0.185	0.737 (0.461, 1.178)	0.202
	Low	246 (24.2%)	0.328 (0.202, 0.534)	<0.001	0.339 (0.203, 0.567)	<0.001
**Personal Covid Experience**	No	428 (42.1%)	1			
	Yes	588 (57.9%)	0.89 (0.664, 1.194)	0.438		
**HIV**	Negative	126 (12.4%)	1			
	Positive	890 (87.6%)	0.729 (0.481, 1.107)	0.138		
**Internet use in the last 30 days**	No	420 (41.4%)	1			
	Yes	594 (58.6%)	1.813 (1.33, 2.472)	<0.001	1.528 (1.089, 2.144)	0.014

Table S6. Primary sources of media information: Whatsapp

Television						
**Variable**	Category	Frequency	Univariate		Multivariate	
			OR (95% CI)	p-value	OR (95% CI)	p-value
**Gender**	Male	508 (50.0%)	1			
	Female	508 (50.0%)	0.904 (0.702, 1..166)	0.438		
**Age**	18-25	368 (36.2%)	1			
	26-39	409 (40.3%)	0.866 (0.641, 1.168)	0.345		
	≥40	239 (23.5%)	0.413 (0.295,0.578)	<0.001		
**Education**	Primary	89 (8.8%)	1			
	Lower Secondary	735 (72.4%)	3.708 (2.314, 5.944)	<0.001	2.92 (1.796, 4.747)	<0.001
	Higher Secondary	117 (11.5%)	5.545 (3.038, 10.124)	<0.001	2.74 (1.446, 5.193)	0.002
	Tertiary	74 (7.3%)	6.778 (3.385, 13.572)	<0.001	2.828 (1.347, 5.935)	0.006
**Economic Status**	High	172 (16.9%)	1			
	Middle	598 (58.9%)	0.783 (0.543, 1.129)	0.191	0.91 (0.616, 1.343)	0.635
	Low	246 (24.2%)	0.462 (0.307, 0.697)	<0.001	0.615 (0.394, 0.96)	0.032
**Personal Covid Experience**	No	428 (42.1%)	1			
	Yes	588 (57.9%)	0.914 (0.707, 1.181)	0.493		
**HIV**	Negative	126 (12.4%)	1			
	Positive	890 (87.6%)	0.786 (0.538, 1.147)	0.211		
**Internet use in the last 30 days**	No	420 (41.4%)	1			
	Yes	594 (58.6%)	3.747 (2.819, 4.982)	<0.001	3.253 (2.399, 4.41)	<0.001

Table S7. Primary sources of media information: Facebook

Television						
**Variable**	Category	Frequency	Univariate		Multivariate	
			OR (95% CI)	p-value	OR (95% CI)	p-value
**Gender**	Male	508 (50.0%)	1			
	Female	508 (50.0%)	0.627 (0.486, 0.81)	<0.001	0.741 (0.56, 0.982)	0.037
**Age**	18-25	368 (36.2%)	1			
	26-39	409 (40.3%)	0.731 (0.55, 0.972)	0.031	0.962 (0.707, 1.307)	0.803
	≥40	239 (23.5%)	0.217 (0.146, 0.324)	<0.001	0.297 (0.195, 0.453)	<0.001
**Education**	Primary	89 (8.8%)	1			
	Lower Secondary	735 (72.4%)	4.045 (2.114, 7.741)	<0.001		
	Higher Secondary	117 (11.5%)	6.736 (3.253, 13.95)	<0.001		
	Tertiary	74 (7.3%)	8.342 (3.826, 18.188)	<0.001		
**Economic Status**	High	172 (16.9%)	1			
	Middle	598 (58.9%)	0.545 (0.387, 0.767)	<0.001	0.605 (0.419, 0.875)	0.008
	Low	246 (24.2%)	0.268 (0.176, 0.409)	<0.001	0.372 (0.236, 0.586)	<0.001
**Personal Covid Experience**	No	428 (42.1%)	1			
	Yes	588 (57.9%)	1.054 (0.814, 1.364)	0.69		
**HIV**	Negative	126 (12.4%)	1			
	Positive	890 (87.6%)	0.518 (0.338, 0.794)	0.003		
**Internet use in the last 30 days**	No	420 (41.4%)	1			
	Yes	594 (58.6%)	3.783 (2.892, 4.948)	<0.001	3.029 (2.281, 4.021)	<0.001
